# Sternal Tuberculosis

**DOI:** 10.4269/ajtmh.19-0200

**Published:** 2019-09

**Authors:** Juan Carlos Cataño, Isabel Cristina Ramirez

**Affiliations:** 1 Infectious Diseases Section, University of Antioquia Medical School, Medellin, Colombia;; 2 Infectious Diseases Section, Pablo Tobon Hospital, Medellin, Colombia

**Figure 1.**
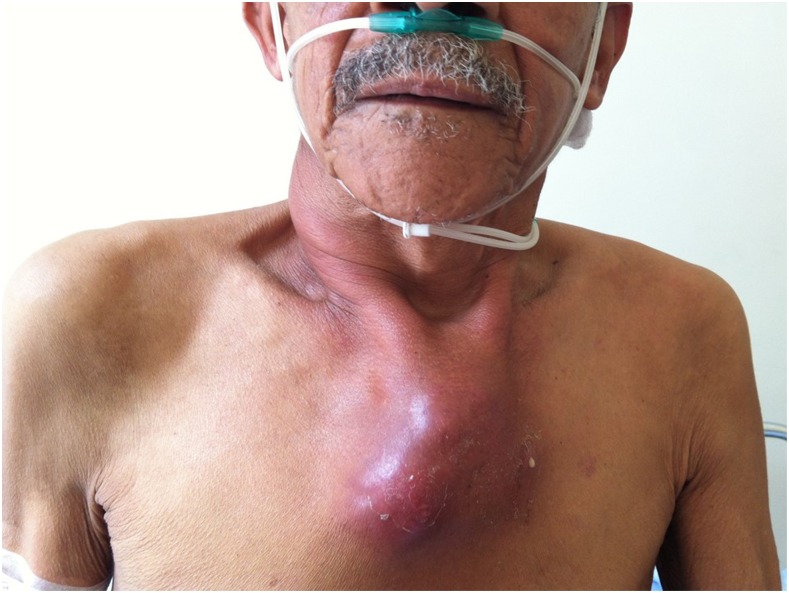
Presternal chest wall mass. This figure appears in color at www.ajtmh.org.

**Figure 2.**
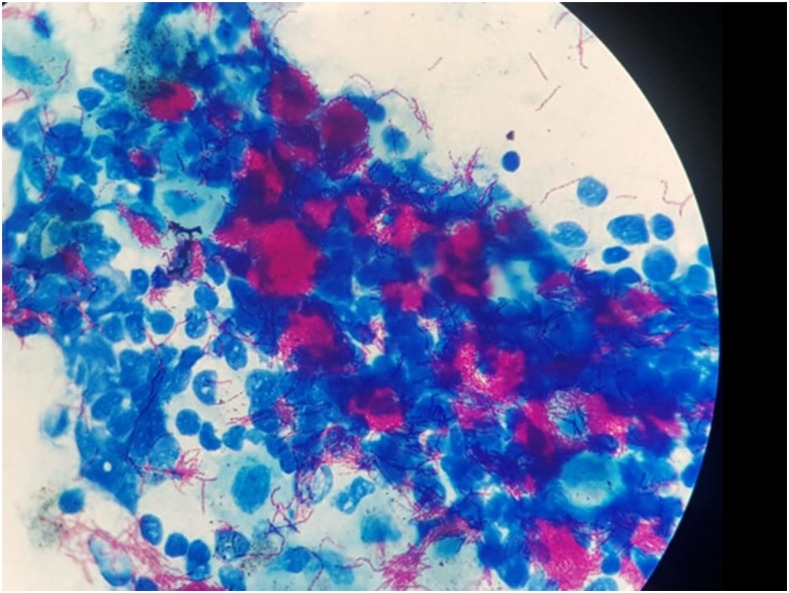
Ziehl-Neelsen stain showing multiple acid-fast bacillae. This figure appears in color at www.ajtmh.org.

A 72-year-old man with diabetes and hypertension presented with 6 months of night sweats, fever, hyporexia, malaise, 12-kg weight loss, and a rapidly growing fluctuant mass over the sternum. He had received various empiric antibiotics (clindamycin, amoxicillin, and trimethoprim/sulfamethoxazole) without improvement. Physical examination showed a cachectic man not in distress. Cervical lymphadenopathy and a 10-cm fluctuant presternal chest wall mass were present; there was a fistula draining non-fetid purulent material (Figure 1). The remaining physical examination was normal. Significant laboratory results include negative routine blood cultures, hyperglycemia (278 mg/dL), elevated glycosylated hemoglobin (7%), mild leukocytosis (11,700 cells/mL), normocytic anemia (10.2 mg/dL), and creatinine (2.2 mg/dL). Computed tomography scan of the chest was reported to show erosion of the anterior cortex of the sternum with thickened and inflamed overlying tissue suggestive of chronic osteomyelitis, with substernal abscess. The patient underwent surgical drainage, which demonstrated abundant caseous material. Histologic examination of the sternal bone showed necrosis, granulomas with multinucleated giant cells, and abundant acid-fast bacilli in the fistular and soft tissue material (Figure 2). Molecular testing (Genotype MTBDR) was positive for *Mycobacterium tuberculosis* sensitive to isoniazid and rifampin. First-line antituberculous treatment was given with good response. Mycobacterial cultures were positive on the clinical specimens. HIV infection and pulmonary tuberculosis (TB) were not found.

Tuberculosis remains a significant global cause of morbidity and mortality, especially in the developing regions. Primary tubercular sternal osteomyelitis is rarely described. A painful mass over the anterior chest wall and signs of soft tissue inflammation and fistula are typical findings, representing delayed diagnosis. Spontaneous sternal fracture, extrasternal spread, and acute inflammatory response (sepsis or septic shock) have also been described.^1,2^ Differential diagnosis includes pyogenic abscess due to *Staphylococcus aureus* and actinomycosis, and where are endemic, histoplasmosis and botryomycosis. Final diagnosis is based on radiological findings and microbiological and histological examination of the debrided tissues, and should include special stains, cultures, and molecular diagnostics. Management is similar to other forms of extrapulmonary TB.^3^
